# Rapid Onset Severe Immune Thrombocytopenia following mRNA COVID-19 Vaccine in a Young Patient

**DOI:** 10.1155/2023/7877536

**Published:** 2023-03-13

**Authors:** Jorge Avila, Huseyin Berk Degirmenci, Pamela Contreras Chavez, Elisabeth M. Battinelli, Jorge Fleisher

**Affiliations:** ^1^Department of Medicine, St. Elizabeth's Medical Center, Tufts University School of Medicine, Boston, MA, USA; ^2^Dana Farber Cancer Institute at St. Elizabeth's Medical Center, Boston, MA, USA; ^3^Division of Hematology, Brigham and Women's Hospital, Harvard Medical School, Boston, MA, USA; ^4^Division of Infectious Diseases, St. Elizabeth's Medical Center, Tufts University School of Medicine, Boston, MA, USA

## Abstract

The coronavirus disease 2019 (COVID-19) pandemic has affected millions of people around the world. Vaccination against COVID-19 has been approved for the following three vaccines in the United States: Pfizer-BioNTech, Moderna, and Janssen. Hematological complications of vaccination have been reported in the literature but remain as a rare phenomenon. We present the case of a patient who developed severe thrombocytopenia within twenty-four hours following the Pfizer-BioNTech vaccination. Commonly encountered differentials including heparin-induced thrombocytopenia and common viral etiologies were ruled out, and other causes such as drug reactions deemed unlikely as the etiology of this presentation after a broad workup. Nucleocapsid antibodies against COVID-19 were found to be positive which indicated that vaccination was at least the second encounter with this virus for our patient, which has been reported previously as the cause of immune thrombocytopenia (ITP), and this might be the culprit for sudden onset. He responded to the first-line ITP treatment with corticosteroids and intravenous immunoglobulin (IVIG) as evidenced by the fast recovery of platelet count and lack of recurrence of thrombocytopenia.

## 1. Introduction

Coronavirus disease 2019 (COVID-19) is an infectious disease caused by the severe acute respiratory syndrome coronavirus-2 (SARS-CoV-2) virus, the causative virus for one of the most significant infections of our era. The United States Food and Drug Administration (US-FDA) has approved multiple vaccines including Pfizer-BioNTech, Moderna, and Janssen against this virus; meanwhile, other countries are currently administrating ChAdOx1 (AstraZeneca) and SinoVac COVID-19 vaccines. Hematological complications including vaccine-induced immune thrombotic thrombocytopenia (VITT) have been reported as a rare but severe adverse event of the COVID-19 vaccine [1]. Exacerbation of chronic immune thrombocytopenia (ITP) has also been published and linked to the administration of these vaccines [2]; however, de novo ITP is considered a much rare complication of vaccination and only a few cases have been reported for this side effect following the Pfizer-BioNTech vaccine to our knowledge.

Hereby, we present the case of a patient who developed severe thrombocytopenia within 24 hours after the administration of the Pfizer-BioNTech vaccine.

## 2. Case Presentation

A 25-year-old male with active intravenous drug use presented to the emergency department from a homeless encampment due to fevers and ill appearance. Upon arrival, he was in septic shock and subsequent cultures grew high-grademethicillin-resistant*Staphylococcus aureus* (MRSA) bacteremia. His initial laboratory work was pertinent for white blood cell count (WBC) of 14.7 × 10^3^/uL, hemoglobin (Hgb) count of 7.9 g/dL, and platelet (PLT) count of 73 × 10^3^/uL. Initial coagulation studies showed normal PT/INR and aPTT. He did not have any history of thrombocytopenia and new onset thrombocytopenia was attributed to sepsis-induced bone marrow suppression. Although he was initially placed on vancomycin for MRSA bacteremia, his antibiotic regimen had to be modified to daptomycin and ceftaroline on hospital day 4 (HD4) given the persistent bacteremia on vancomycin and relatively elevated minimal inhibitory concentration (MIC). Further infectious workup showed negative human immunodeficiency virus (HIV) and EpsteinBarr virus (EBV) serologies and a positive hepatitis C viremia, which was previously documented. Echocardiogram revealed large vegetation (2.8 × 3.2 cm) on the anterior leaflet of the tricuspid valve with mild tricuspid regurgitation (TR). His repeat echocardiogram after 7 days of antibiotic therapy showed a size change of vegetation to 2.78 × 1.61 cm (from 2.8 × 3.2 cm) and a worsening of TR to moderate-severe from mild. Computed tomography (CT) of the chest showed innumerable cavitary lesions consistent with septic emboli along with multifocal abscesses. He underwent chest tube placement for management of loculated pulmonary effusion on hospital day 8 and fluid analysis was consistent with empyema with positive cultures for MRSA. His blood cultures finally cleared on day 10, and his repeat laboratory work on day 10 showed a WBC of 12.0 × 10^3^/uL, Hgb of 9.4 g/dL, and a PLT count of 174 × 10^3^/uL.

During the following days, his PLT count ranged between 146 × 10^3^/uL and 216 × 10^3^/uL with a PLT count of 156 × 10^3^/uL on day 15. He received mRNA COVID-19 Pfizer vaccination on the afternoon of hospital day 15 (HD15) and his platelet count the next morning was 0 × 10^3^/uL which was confirmed by repeating the analysis several times with citrated tubes. The peripheral smear was negative for any clumping or hemolysis, and it showed large platelets. Coagulation studies revealed fibrinogen of 384 mg/dL (187–446), PT of 12.4 seconds (9.3–12.1), INR of 1.2 (0.9–1.2), aPTT of 31.9 seconds (23.9–32.8), and D-dimer of 5.90 ug/mlFEU (<0.50).

We also calculated the 4T score in this patient and it was found to be 5, which is why prophylactic subcutaneous enoxaparin was discontinued, and a heparin-induced thrombocytopenia panel was sent which later showed negative platelet factor-4 (PF-4) antibodies and negative serotonin release assay (SRA).

**Figure 1:**
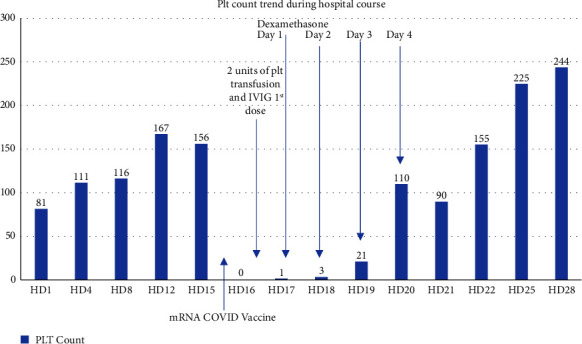
Platelet count during hospital stay incorporating COVID vaccination and treatment timeline. PLT: platelets; HD: hospital day; IVIG: intravenous immunoglobulin.

Infectious disease (ID) service was consulted and ceftaroline was stopped given its link to thrombocytopenia although it was deemed unlikely to be the culprit in this case given the very abrupt drop, single-digit platelet nadir, and atypical temporality as the patient had been on the medication for more than a week. Two units of platelets were transfused without appropriate response as the posttransfusion platelet count was 1 × 10^3^/uL. Given no response to platelet transfusions, the possibility of immune thrombocytopenic purpura (ITP) with ongoing destruction was raised by the hematology service, and he was initiated on intravenous immunoglobulin (IVIG). The patient developed an adverse reaction after the first dose of IVIG which was attributed to the transfusion-related acute lung injury (TRALI); thus, he could not receive the second dose of IVIG. Subsequently, dexamethasone 40 mg daily was started for a total of four days. His PLT count started improving after dexamethasone administration and the daily PLT count trend was as follows: 0 (day 17); 1, 3, 21, 110 (day 20); 90, 155 × 10^3^/uL (day 22), and it remained normal afterwards (Figure 1). Coronavirus nucleocapsid protein antibodies (anti-N abs) were sent as per ID request for testing and returned positive which proved suspected previous exposure to SARS-CoV-2 before the vaccine, since nucleocapsid antibodies are triggered only by the infection, whereas spike antibodies are triggered by infection and/or vaccination. This indicated that mRNA COVID-19 vaccination was at least his second encounter with SARS-CoV-2 antigens which likely made a very rapid onset immunological process possible.

His thrombocytopenia did not recur afterwards, and he is currently completing his daptomycin course with a plan to follow up with cardiothoracic surgery for valve surgery evaluation.

## 3. Discussion

In the placebo-controlled, observer-blinded, randomized trial of the Pfizer-BioNTech vaccine, from the 43448 patients who received the vaccine, only the following four severe adverse events were reported: shoulder injury related to vaccine administration, right axillary lymphadenopathy, paroxysmal ventricular arrhythmia, and right leg paresthesia [3]. Data from the Vaccine Adverse Event Reporting System (VAERS) presented in 2022 report 77 cases of de novo ITP in patients that received either the Pfizer-BioNTech (BNT162b2) (37 patients) or Moderna (mRNA-1273) (40 patients), with a mean of 8 days to symptom onset after the administration of either of these vaccines (the earliest being 3.2 days) [4]. This time frame was also similar to the reports from the AstraZeneca vaccine that also showed an incidence of 8 ITP cases per million following the first dose [5]. However, it has also been shown that the onset of symptoms can occur as early as one day after vaccination [6].

In a series of cases with thrombocytopenia occurring within 2 weeks of SARS-CoV-2 vaccination and leading to the development of ITP (identified in the VAERS with a last search of 2/5/2021), they described patients presenting with symptoms such as petechiae, bruising, mucosal bleeding, chest pain, and hemorrhage, and it was reported that their clinical presentations along with their favorable response to standard ITP treatments such as IVIG and corticosteroids suggested an antibody-mediated platelet process such as ITP. Also, most of these patients did not respond to platelet transfusions (similar to our patient from this case report), confirming a pattern suggestive of ITP [6]. In our patient's clinical presentation, the platelet drop onset was very rapid (within 24 hours) and one could argue possible predisposing etiologies such as drug-induced and sepsis-induced thrombocytopenia from bone marrow suppression. He initially presented with sepsis from tricuspid valve endocarditis and was started on vancomycin, followed by daptomycin and ceftaroline for the antibiotic regimen which led to resolution of initial thrombocytopenia from 78 × 10^3^/uL up to 146 × 10^3^/uL, with the sudden drop of PLT count to 0 the next day after mRNA COVID-19 Pfizer vaccination. As known, several infections have been associated with ITP including but not limited to *Helicobacter pylori*, hepatitis C virus, and HIV [7]. Our patient did have a history of chronic hepatitis C; however, his platelet count was normal throughout the hospital course until the rapid drop following the vaccine. Previous data have also identified daptomycin-induced ITP as a probable entity of thrombocytopenia [8]; however, in our patient, this drug was continued during his hospitalization course and was maintained even after ITP resolution, making it unlikely to be the cause of the ITP. It was difficult to determine if any of these conditions were contributing factor(s) to his rapid onset of ITP but deemed unlikely to be a causative factor(s) in his case.

The leading theory to explain the phenomenon of ITP is that the viral antigens cross-react with normal platelet antigens in molecular mimicry, causing the destruction of these cells [9]. Reactions can occur shortly after exposure to the trigger. For example, if the patient has been previously sensitized to an antigen, the onset of thrombocytopenia can be as early as hours after reexposure [10]. This seems to be the case in our patient as he tested positive for anti-N abs, which is the standard assay to determine previous infection [11], confirming prior COVID-19 exposure. It is important to note that anti-N abs are not elicited by COVID-19 vaccines that target the spike protein, including all vaccines used in the United States [12].

Other authors propose that the mRNA component of the Pfizer-BioNTech vaccine is unlikely to cause an allergic reaction by itself, but rather the modified RNA trace impurities that are present in this vaccine could explain thrombocytopenia. It is also reported that there is a possibility that these aberrant proteins could initiate an immune response that would lead to this adverse reaction [13].

Vaccines prepared from dead bacteria or viruses are potent stimulators of the immune system in general, and many adjuvants (DNA, lipids, and aluminum salts) are commonly added to other vaccines to enhance the immune system. The potent immune activator is mRNA, as this is the nanoparticle lipid component of the COVID-19 vaccines [14]. Recent publications show that mRNA is taken up by cellular RNA receptors leading to the upregulation of toll-like receptors (TLR) 7 and 8 followed by the activation and maturation of immune cells and secretion of cytokines and chemokines [15]. Nonetheless, data show that a second COVID-19 vaccination using a different mRNA vaccine can be considered safe for selected patients who had experienced de novo ITP after the administration of a first dose [16].

COVID-19 vaccination has been linked to multiple adverse effects and thrombocytopenia is considered one of them. However, de novo ITP is not as common as VITT or recurrence of chronic ITP. Data suggest that although it can occur, most of the cases are mild with no life-threatening complications and respond to first- or second-line therapies used for ITP although a few cases might necessitate third-line treatments. ITP is not a contraindication for COVID-19 vaccination, and the rarity of these events and excellent outcomes for patients should not change views regarding the benefits provided by immunization [17].

## Data Availability

The data used to support the findings of the study can be obtained from the corresponding author upon request.

## References

[1] Chou S. C., Chang Y. C., Liao C. K. (2022). New presentations and exacerbations of immune thrombocytopenia after coronavirus disease 2019 vaccinations: the Taiwan experience. *Platelets*.

[2] Ogai A., Yoshida R., Yuasa C., Chin K., Fujimaki K., Nakajima H. (2022). Acute immune thrombocytopenia following SARS-CoV-2 vaccination in chronic ITP patients and a healthy individual. *International Journal of Hematology*.

[3] Polack F. P., Thomas S. J., Kitchin N. (2020). Safety and efficacy of the BNT162b2 mRNA covid-19 vaccine. *New England Journal of Medicine*.

[4] Lee E. J., Beltrami-Moreira M., Al-Samkari H. (2022). SARS-CoV-2 vaccination and ITP in patients with de novo or preexisting ITP. *Blood*.

[5] Gordon S. F., Clothier H. J., Morgan H. (2021). Immune thrombocytopenia following immunisation with vaxzevria ChadOx1-S (AstraZeneca) vaccine, victoria, Australia. *Vaccine*.

[6] Lee E. J., Cines D. B., Gernsheimer T. (2021). Thrombocytopenia following pfizer and Moderna SARS-CoV-2 vaccination. *American Journal of Hematology*.

[7] Cines D. B., Bussel J. B., Liebman H. A., Luning Prak E. T. (2009). The ITP syndrome: pathogenic and clinical diversity. *Blood*.

[8] Grégoire C., Brumpt C., Loirat D. (2012). A case of daptomycin-induced immune thrombocytopenia. *Antimicrobial Agents and Chemotherapy*.

[9] Kenney A., Adhikari A. (2021). Immune thrombocytopenia in a 68-year-old woman after COVID-19 vaccination. *Clinical Case Reports*.

[10] Kenney B., Stack G. (2009). Drug-induced thrombocytopenia. *Archives of Pathology & Laboratory Medicine*.

[11] Krutikov M., Palmer T., Tut G. (2022). Prevalence and duration of detectable SARS-CoV-2 nucleocapsid antibodies in staff and residents of long-term care facilities over the first year of the pandemic (VIVALDI study): prospective cohort study in England. *The Lancet Healthy Longevity*.

[12] Follmann D., Janes H. E., Buhule O. D. (2022). Antinucleocapsid antibodies after SARS-CoV-2 infection in the blinded phase of the randomized, placebo-controlledmRNA-1273 COVID-19 vaccine efficacy clinical trial. *Annals of Internal Medicine*.

[13] Malayala S. V., Papudesi B. N., Sharma R., Vusqa U. T., Raza A. (2021). A case of idiopathic thrombocytopenic purpura after booster dose of BNT162b2 (Pfizer-Biontech) COVID-19 vaccine. *Cureus*.

[14] Ndeupen S., Qin Z., Jacobsen S., Estanbouli H., Bouteau A., Igyártó B. Z. (2021). The mRNA-LNP platform’s lipid nanoparticle component used in preclinical vaccine studies is highly inflammatory. *bioRxiv*.

[15] Edwards D. K., Jasny E., Yoon H. (2017). Adjuvant effects of a sequence-engineered mRNA vaccine: translational profiling demonstrates similar human and murine innate response. *Journal of Translational Medicine*.

[16] Chanut M., Jaidi R., Kohn M. (2022). Successful mRNA SARS-Cov-2 vaccine rechallenge after a first episode of immune thrombocytopenic purpura. *Platelets*.

[17] Saluja P., Amisha F., Gautam N., Goraya H. (2022). A systematic review of reported cases of immune thrombocytopenia after COVID-19 vaccination. *Vaccines*.

